# Mr. Edwards, on Ulcers

**Published:** 1801-03

**Authors:** W. Edwards

**Affiliations:** Member of the College of Surgeons in London, &c. Taunton Barracks


### *To the Editors of the Medical and Phyſical Journal.*

Gentlemen,

On peruſing a paper of Mr. Ballard, ſurgeon in the Royal Navy, inſerted in No. xxi. of your valuable Journal, on a particular ſpecies of Ulcer; my having been for ſome years an aſſiſtant ſurgeon to the Royal Naval Hoſpital at Haſlar, enables me to give the moſt ſucceſsful mode I could find of treating that ſpecies of ulcer at the above Hoſpital. When thoſe kind of patients were firſt received, they were well waſhed with warm water and ſoap, and if the general debility was not very great, had a doſe of ſome laxative medicine; after its operation, an opiate, the ulcer dreſſed with a large emollient cataplaſm, compoſed of linſeed meal and fine pollard, and before each dreſſing fomented with a ſtrong decoction of Peruvian bark and poppy heads for the ſpace of half an hour, the dreſſing renewed in general twice a day; but if the ulcer was very foul, with great diſcharge and extenſive ſloughs, it was dreſſed oftener, till it put on a healthy granulating appearance. During the above time, the patient took daily a pint or ſometimes more of a decoction of Peruvian bark, after its having gone through the vinous fermentation; the common drink was lemonade; and whilſt in great pain, one grain of opium was given twice or thrice a day, with a double doſe at night; the ſymptomatic fever which generally attended, was commonly abated by the above means in a few days; wine and porter were alſo given occaſionally to ſuch caſes as required: The ulcer being by the above means brought to a clean healthy appearance, ſlight ſtimulating applications, as cuprum vitriolatum, argentum nitratum, &c. were applied round its edges, in order to accelerate its cicatrization, and a pledget of ſperm. ceti cerate very thinly ſpread upon lint put over the whole ulcer, with a ſoft linen compreſs over that, and a moderately tight bandage over all; the dreſſing renewed once or twice in the twenty-four hours, according to the quantity of diſcharge. After being brought to a ſmall ſize, it often proved very obſtinate to heal over compleat; to accelerate which, Mr. Baynton's very excellent mode of treating ulcerated legs, &c. by adheſive plaſters, proved very ſucceſsful, when ſuch happened to be in any of the extremities, and the inferior extremities are the chief ſubject of ſuch ulcers.—During the cure, the patients lived chiefly on animal food and vegetables, with good ſoup, which, no doubt, contributed in a great degree to mend the general health, as the patients' countenances on their firſt admiſſion always appeared wan and dejected, which was a proof that digeftion and chylification went on but imperfectly; therefore, I have every reafon to believe, if the nitrous fumigation is perfifted in daily on fhip board, particularly in the Sick Bay, with frequently bathing the men in the cold or tepid falt water bath, would tend very much to invigorate their fyftems, better enable the organs of digeftion to perform their office, and prevent the contagion of thofe ulcers, as it is evident their contagion and obftinacy proceed from the vitiated ftate of the body; for if any efcharotic is applied to them before the general habit is mended, it corrodes the mufcular fubftance, whereas, if the habit of body is good, it will produce fine florid granulations.

During the whole of laft winter, I was attending the fick and wounded Ruffian troops, that were received into the temporary general Hofpital at N. Yarmouth, after being wounded in Holland. From the number we had there, they were obliged to be ftowed very clofe together in low ceiled houfes, and fome of thofe houfes fituated in very clofe, fwampy fituations, where they could get but little light, and lefs good air; the fœtor of their wounds, as may be fuppofed, muft be great, and the men being naturally filthy in their perfons, muft make it ftill worfe; however, by the ftrict attention paid to the nitrous fumigation, and frequent ufe of the falt water bath, by order of Dr. Scot, Affiftant Infpector of Hofpitals, who fuperintended the medical department there, every appearance of contagion was prevented. In fome of the convalefcent quarters, a few cafes of fynochus broke out; but by feparating them, and perfevering in the fumigation, it went no farther. I applied the nitrous gas to foul floughing ulcers, by means of a tube from the top of a patent fumigating lamp, and it had the effect of clearing them; but the foulnefs and floughing returned in few dreffings after the gas was omitted; thefe ulcers were afterwards brought to a clean healthy ftate by the application of the common cataplafm, made with fea in lieu of common water, and applied cold, without any intervening fubftance, except in fome cafes, where, from the irritability of the ulcer it could not be borne; in thofe cafes dry lint anfwered the intention better than greafy applications; in fome of them I was obliged to continue the poultice till the ulcer compleatly healed, for when it was left off the ulcer relapfed, but renewing the poultice foon counteracted its regeneration. In fome of thofe vitiated ulcers, the common poultice, with the cortical part of a frefh carrot fcraped fine, and applied over it, had alfo a very good effect; I gave no medicine internally to them, except an opiate at night, whilft the appetite kept good, and a good nourifhing diet of animal food boiled down down into ſoup, with plenty of vegetables and Scotch barley or oatmeal to thicken it.

I alſo applied the nitrous fumigation in caſes of ſynochus, by placing two fumigating lamps, one on each ſide of the patient's bed: The effects were in a ſhort time viſible, he expreſſed that a glow was coming over him; the pulſe raiſed fifteen ſtrokes in the minute in the ſpace of two hours, and a free perſpiration followed, and then he fell into a ſound ſleep for ſome time. When he awoke, he aſked for drink; and by perſevering in the fumigation and the uſe of bark and opium, he recovered. If you think the above ſketches worth inſerting in your Journal, you will oblige a conſtant reader by ſo doing. I am, &c.

W. EDWARDS,
Member of the College of Surgeons in London, &c.

Taunton Barracks, Jan. 8th, 1801.

---

